# In depth functional characterization of human induced pluripotent stem cell-derived beta cells *in vitro* and *in vivo*

**DOI:** 10.3389/fcell.2022.967765

**Published:** 2022-08-17

**Authors:** Federica Fantuzzi, Sanna Toivonen, Andrea Alex Schiavo, Heeyoung Chae, Mohammad Tariq, Toshiaki Sawatani, Nathalie Pachera, Ying Cai, Chiara Vinci, Enrico Virgilio, Laurence Ladriere, Mara Suleiman, Piero Marchetti, Jean-Christophe Jonas, Patrick Gilon, Décio L. Eizirik, Mariana Igoillo-Esteve, Miriam Cnop

**Affiliations:** ^1^ ULB Center for Diabetes Research, Université Libre de Bruxelles, Brussels, Belgium; ^2^ Endocrinology and Metabolism, Department of Medicine and Surgery, University of Parma, Parma, Italy; ^3^ Institut de Recherche Expérimentale et Clinique, Pôle d’Endocrinologie, Diabète et Nutrition, Université Catholique de Louvain, Brussels, Belgium; ^4^ Department of Clinical and Experimental Medicine, University of Pisa, Pisa, Italy; ^5^ Division of Endocrinology, Erasmus Hospital, Université Libre de Bruxelles, Brussels, Belgium

**Keywords:** aggregate, beta cell, human induced pluripotent stem cell, insulin secretion, islet, microwell

## Abstract

*In vitro* differentiation of human induced pluripotent stem cells (iPSCs) into beta cells represents an important cell source for diabetes research. Here, we fully characterized iPSC-derived beta cell function *in vitro* and *in vivo* in humanized mice. Using a 7-stage protocol, human iPSCs were differentiated into islet-like aggregates with a yield of insulin-positive beta cells comparable to that of human islets. The last three stages of differentiation were conducted with two different 3D culture systems, rotating suspension or static microwells. In the latter, homogeneously small-sized islet-like aggregates were obtained, while in rotating suspension size was heterogeneous and aggregates often clumped. *In vitro* function was assessed by glucose-stimulated insulin secretion, NAD(P)H and calcium fluctuations. Stage 7 aggregates slightly increased insulin release in response to glucose *in vitro*. Aggregates were transplanted under the kidney capsule of NOD-SCID mice to allow for further *in vivo* beta cell maturation. In transplanted mice, grafts showed glucose-responsiveness and maintained normoglycemia after streptozotocin injection. *In situ* kidney perfusion assays showed modulation of human insulin secretion in response to different secretagogues. In conclusion, iPSCs differentiated with equal efficiency into beta cells in microwells compared to rotating suspension, but the former had a higher experimental success rate. *In vitro* differentiation generated aggregates lacking fully mature beta cell function. *In vivo*, beta cells acquired the functional characteristics typical of human islets. With this technology an unlimited supply of islet-like organoids can be generated from human iPSCs that will be instrumental to study beta cell biology and dysfunction in diabetes.

## Introduction

Diabetes develops when the amount of insulin secreted fails to meet the body’s metabolic demands. The cellular and molecular mechanisms underlying pancreatic beta cell failure vary across different types of diabetes (Nolan et al., 2011; Herold et al., 2013; Cnop et al., 2017). Human pluripotent stem cells provide an unlimited cell source to produce beta cells for mechanistic and therapeutic studies and for cell replacement in diabetes (Balboa et al., 2019). During the past decade the methods to differentiate pluripotent stem cells into beta cells have improved substantially (Pagliuca et al., 2014; Rezania et al., 2014; Russ et al., 2015; Petersen et al., 2018; Balboa et al., 2019). Beta cells are generated by stimulating stem cells with a series of small molecules and growth factors that guide them into pancreatic developmental steps (Petersen et al., 2018; D'Amour et al., 2005; Jennings et al., 2015; Pan and Wright, 2011).

An important advance in disease modeling with stem cells occurred with the development of three-dimensional (3D) organotypic culture systems (Liu et al., 2018). The 3D environment allows better cell-to-cell communication and correct cellular polarization. Correct islet architecture and beta cell polarization are crucial for beta cell functionality. *In vitro* organoid systems require a platform to induce self-organization and lineage specification. Natural scaffold-based strategies rely on laminin-rich Matrigel, pure laminins, collagens or other biomaterials, which favor cell differentiation and functionality. Several reports have described beta cell differentiation using natural or synthetic scaffolds (Kuo et al., 2017; An et al., 2018; Candiello et al., 2018). However, pure extracellular matrix proteins are costly, limiting large-scale organoid production. For large-scale production, beta cells can be differentiated in dynamic suspension cultures or bioreactors but organoid formation and size are not well controlled (Pagliuca et al., 2014; Mihara et al., 2017). Uncontrolled aggregation easily results in too large organoids, which undergo central necrosis and hamper beta cell maturation. Smaller organoids can coalesce into bigger unorganized cell clumps, leading to failed experiments.

Many laboratories have reported successful differentiation of insulin-releasing beta cells *in vitro* and *in vivo*. In these studies, functional human stem cell-derived beta cells were generated by sorting more mature beta cells in order to generate beta cell-enriched clusters (Nair et al., 2019) or by modulating signaling in the last stages of differentiation, e.g., TGF-beta (Velazco-Cruz et al., 2019) and WNT4 signaling (Yoshihara et al., 2020). Despite improvements in human stem cell differentiation into beta cells, the methods remain technically challenging and limited by poor reproducibility. In this study we report a scalable and easy technique to generate homogeneously small-sized islet-like organoids from human induced pluripotent stem cells (iPSCs) with great experimental success rate. We characterize in depth the function of iPSC-derived beta cells *in vitro* and *in vivo* after transplantation.

## Material and methods

### Ethical approval

*In vivo* studies were performed with the approval of the Commission d’Ethique et du Bien Être Animal (CEBEA), Medical Faculty, Université Libre de Bruxelles. The CEBEA follows the European Convention for the Protection of Vertebrate Animals used for Experimental and other Scientific Purposes (European Treaty Series No.123). Human pancreata, not suitable for transplantation, were collected from non-diabetic brain-dead organ donors with the approval of the Ethical Committee of the University of Pisa, Italy, after signed informed consent by next-of-kin (Marchetti et al., 2018). Human iPSCs were reprogrammed from skin fibroblasts with approval by Ethical committees, see below. The differentiation of iPSCs into beta cells was approved by the Ethical Committee of the Erasmus Hospital, Université Libre de Bruxelles, reference P2019/498.

### Cell culture

Human clonal EndoC-βH1 cells (kindly provided by Raphael Scharfmann, Cochin Institute, Paris, France) (Ravassard et al., 2011) were cultured in DMEM (ThermoScientific) as described (Grieco et al., 2014; Brozzi et al., 2015). Human islets (from 9 non-diabetic donors, age 67 ± 10 years, BMI 26.9 ± 3.2 kg/m^2^, Supplementary Table S1) were isolated by collagenase digestion and density gradient purification in Pisa (Marchetti et al., 2018; Marselli et al., 2020) and cultured in Brussels as described (Santin et al., 2011). The beta cell purity of the human islets, determined by insulin immunofluorescence (Cunha et al., 2008), was 55.2 ± 22.0%.

### Human induced pluripotent stem cell differentiation into beta cells

The human iPSC line HEL115.6 (Cosentino et al., 2018; De Franco et al., 2020) was reprogrammed from skin fibroblasts (with approval by the Ethics Committees of the Hospital District of Helsinki and Uusimaa (no. 423/13/03/00/08) and Erasmus Hospital) by Cytotune iPSC reprogramming (Life technologies) (Trokovic et al., 2014) at Biomedicum Helsinki Stem Cell Center (Cosentino et al., 2018). The human iPSC line 1023A was kindly provided by Dieter M Egli, University of Columbia, and the H1 human embryonic stem cells by Timo Otonkoski, University of Helsinki. ULBi001.BJ.6 was reprogrammed with Sendai virus at ULB Center of Diabetes Research as described in (De Franco et al., 2020). Karyotype, embryoid body assay, pluripotency marker expression and colony morphology validated iPSC pluripotency (Supplementary Figure S1). iPSCs were cultured in Matrigel (Corning BV, Life Sciences) coated plates in E8 medium (Life Technologies) and passaged with 0.5 mmol/L EDTA (Life Technologies) twice weekly. Absence of *mycoplasma* was verified monthly using MycoAlert *Mycoplasma* Detection (Lonza). For beta cell differentiation we used a stepwise protocol (Cosentino et al., 2018; De Franco et al., 2020) modified from published protocols (Pagliuca et al., 2014; Rezania et al., 2014; Saarimaki-Vire et al., 2017). iPSCs were washed with 0.5 mmol/L EDTA, incubated with Accutase (Capricorn Scientific) for 3–8-min and seeded at 1.5–2.5 million cells/3.5-cm Matrigel-coated wells in E8 medium containing 5 μmol/L ROCK inhibitor (Y-27632 dihydrochloride, StemCell technologies). Twenty-four hours later, cells reached confluency and were differentiated in Matrigel-coated wells until the end of stage 4 (St4), after which cells were detached by washes with 0.5 mmol/L EDTA and 5–8-min incubation with Accutase at 37**°**C. After 3-min centrifugation at 250 *g*, cells were resuspended in medium with 10 μmol/L ROCK inhibitor and plated either in suspension or in microwells (AggreWell400, StemCell technologies) at a density of 750 cells/microwell unless otherwise indicated. Basal and differentiation media composition are described in (Cosentino et al., 2018) and Supplementary Tables S2, S3. A detailed differentiation protocol is provided in the Supplementary Methods section.

### RNA extraction, reverse transcription and real-time PCR

RNA was extracted with Poly(A)^+^-RNA oligo-dT 25-coated polystyrene Dynabeads (Life Technologies) following the manufacturer’s instructions. RNA was reverse transcribed using Reverse Transcriptase Core Kit (Eurogentec). Real-time PCR was performed using IQ SYBR Green Supermix on CFX Connect (Bio-Rad) or Rotor-Gene Q cycler (Qiagen). Expression values were corrected for the geometric mean of reference genes *GAPDH* and *ACTB*. Primer sequences are provided in Supplementary Table S4.

### Dispersion of aggregates

Aggregates were washed twice with 0.5 mmol/L EDTA, exposed for 8-min to Accumax (Sigma-Aldrich) and dispersed by repeated pipetting as described (Lytrivi et al., 2021). Knockout serum (Gibco) was added to smother the dissociation. Cells were pelleted and resuspended in medium supplemented with 10 μmol/L ROCK inhibitor for overnight culture.

### Immunofluorescence

Cells were fixed in 4% paraformaldehyde for 15–20-min, permeabilized with 0.5% triton-X100, blocked with UltraV block (ThermoScientific) for 10-min and incubated with primary antibodies diluted in 0.1% Tween in PBS for 3 h at room temperature or overnight at 4°C. Following 30–60-min incubation with secondary antibodies at room temperature samples were mounted with Vectashield with DAPI (Vector Laboratories) and covered with glass coverslips. Antibodies are provided in Supplementary Table S5.

### Flow cytometry

Dispersed aggregates were resuspended in PBS containing “Zombie Aqua” dye (BioLegend) and incubated 20-min in the dark to detect live and dead cells. Cells were fixed and permeabilized, incubated with conjugated antibodies for 2 h at room temperature in Perm/Wash buffer (BD Cytofix/Cytoperm, BD Biosciences). After washes, cells were analyzed using FACSCanto II or LSRFortessa X-20 cytometers (BD Biosciences) and FlowJo software (Tree Star). Antibodies are provided in Supplementary Table S6.

### *In vitro* insulin secretion, insulin and proinsulin content

Twenty aggregates were washed with glucose-free Krebs buffer (Univercell Biosolutions, Toulouse, France), pre-incubated in 1.6 mmol/L glucose Krebs for 30-min, exposed to 2.8, 16.7 or 16.7 mmol/L glucose plus 10 μmol/L forskolin for 1 h and supernatant was collected for human insulin ELISA (Mercodia, Uppsala, Sweden). Cellular (pro)insulin was extracted using acid ethanol (95% ethanol, 5% 12N hydrochloric acid) and quantified by ELISA (Mercodia). Insulin secretion and aggregate (pro)insulin content were normalized to total protein content, measured by protein assay dye (Bio-Rad). For NADPH and intracellular calcium measurements, aggregates were preincubated for 40-min in Krebs containing 0.5 mmol/L glucose. Batches of 10 aggregates were incubated for 1 h at 37°C in 1 ml Krebs containing 2 or 20 mmol/L glucose with or without 25 μmol/L gliclazide. Medium was collected for insulin assay and data normalized to DNA content. For perifusion experiments, 300–500 aggregates were perifused at 37°C, at a flow rate of 0.5 ml/min. After an initial 20-min equilibration with the first solution, they were challenged by various test solutions and effluent was collected every 4-min. Insulin was assayed with home-made radioimmunoassay (Gilon et al., 1993).

### NAD(P)H and intracellular calcium measurements

To measure changes in intracellular calcium concentration ([Ca^2+^]_i_), aggregates were loaded with Fura-2 LR acetoxymethyl ester for 2 h at 37°C in culture medium (non-starved) or Krebs containing no glucose (glucose-starved). They were then perifused with Krebs (flow rate ∼1 ml/min) at 37°C in a temperature-controlled chamber placed on the stage of an inverted microscope. The Fura-2 LR fluorescence ratio (λ_ex_ 340/380 nm; λ_em_ 510 nm) was acquired every 5 s as described (Khaldi et al., 2004). For NAD(P)H measurements, non-starved or 2-h glucose-starved aggregates were perifused as above and NAD(P)H autofluorescence (λ_ex_ 360 nm; λ_em_ 470 nm) was acquired every 10 s (Khaldi et al., 2004).

### iPSC-derived beta cell transplantation

NOD.CB17*-Prkdc* ^*scid*^/NCrCrl (Charles River, UK, purchased at age 6-to-8 weeks) were housed in a specific pathogen-free (SPF) animal facility, Université Libre de Bruxelles. Mice were housed at 21°C, in a 12 h light/dark cycle, with *ad libitum* access to regular chow and water. Transplantations were performed in 8-to-12-week-old male mice (average weight 30.5 ± 0.9 g). Mice were anesthetized with intraperitoneal Ketamine (Nimatek, Dechra, 100 mg/kg)/Xylazine (Rompun, Bayer, 5 mg/kg) injection and aggregates were transplanted under the kidney capsule using a 10 µL precision pipet. Paracetamol (100 mg/L drinking water) was given as analgesic 1 day prior to transplantation and for 10 days after. Seven, 14 and 20–21 weeks after transplantation, mice underwent intraperitoneal glucose tolerance tests (IPGTTs), performed as described (Igoillo-Esteve et al., 2020). After 16-h fast, 2 mg glucose per g body weight was administered. Glycemia was measured using a glucometer (Accu-Chek Aviva Nano, Roche) before (0) and 15, 30, 60, 90 and 120 min after injection. Mouse weight, appearance and mobility were monitored to ensure their welfare. Blood was collected from the tail vein at 0, 30, 60 and 90 min and plasma separated by centrifugation at 3,000 rcf for 20-min at 4°C. C-peptide levels were quantified using human ultrasensitive C-peptide ELISA (Mercodia). At 21–23 weeks after transplantation, mice were injected with a single dose of streptozotocin (200 mg/kg) to selectively ablate mouse beta cells. One week later, mice underwent nephrectomy to retrieve the graft or they were killed by cervical dislocation for kidney perfusion.

### *In situ* kidney perfusion

The iPSC-beta cell grafted kidney was perfused *in situ* at 37°C at 1 ml/min flow rate in a single-pass circuit. A ligature was performed at the level of the abdominal aorta above the coeliac trunk, a catheter inserted in the abdominal aorta and the venous effluent collected by another catheter inserted in the renal vein. To avoid coagulation, the engrafted kidney was first perfused with 1 ml heparinized (50 IU/ml) PBS. After an initial 20-min equilibration with basal perfusion solution, the effluent was collected every 4-min. Insulin was assayed by radioimmunoassay (Gilon et al., 1993).

### Statistical analysis

Sample size calculations are described in Supplementary Methods. Data are shown as violin plots (truncated) of the indicated number (n) of independent experiments, defined as one iPSC-beta cell differentiation or one human islet preparation, shown as individual data points. Dotted line represents the median. Paired two-way ANOVA or mixed effects model analysis (the latter in case of a missing value) were applied. If not specified otherwise, the Bonferroni correction was applied for multiple comparisons.

## Results

### Microwell culture produces standardized human beta cell aggregates with high success rate

**FIGURE 1:**
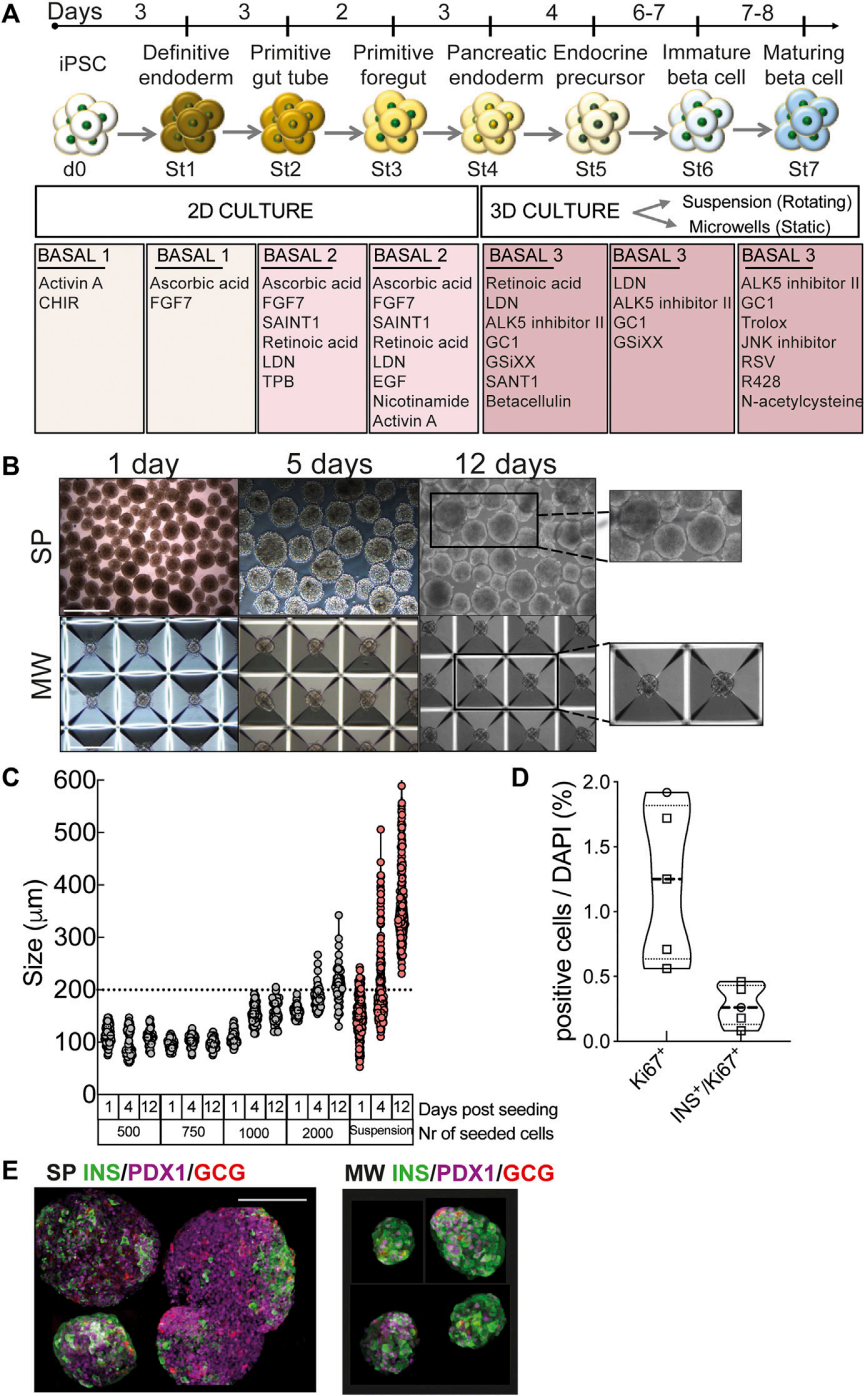
Human iPSC differentiation into beta cells. **(A)**, Human iPSCs were differentiated into beta cells following a 7-stage stepwise protocol. Until stage 4 (St4) cells were cultured in 2D and then transferred to 3D culture, either in suspension (SP) or microwells (MW). Media specification is shown in boxes. **(B)**, Morphology and size of aggregates differentiating into beta cells in rotating suspension (upper panels) or static microwells (bottom panels) at 1, 5 and 12 days post-detachment from 2D culture. Scale bar is 400 µm. **(C)**, Diameter of the aggregates generated with 500, 750, 1000 or 2000 cells per microwell. Diameters were measured at day 1, 4 and 12 post-detachment from 2D culture. **(D)**, Quantification of immunochemical analysis of total or insulin-positive cells (co-)expressing the proliferation marker Ki67 (n = 5). **(E)**, Microwell and suspension aggregates stained for insulin (green), glucagon (red) and PDX1 (purple). Scale bar is 100 µm. Symbols represent different cell models (Hel115.6, circles; 1023A, squares). N, number of independent experiments, defined as one iPSC-beta cell differentiation, shown as individual data points.

**FIGURE 2:**
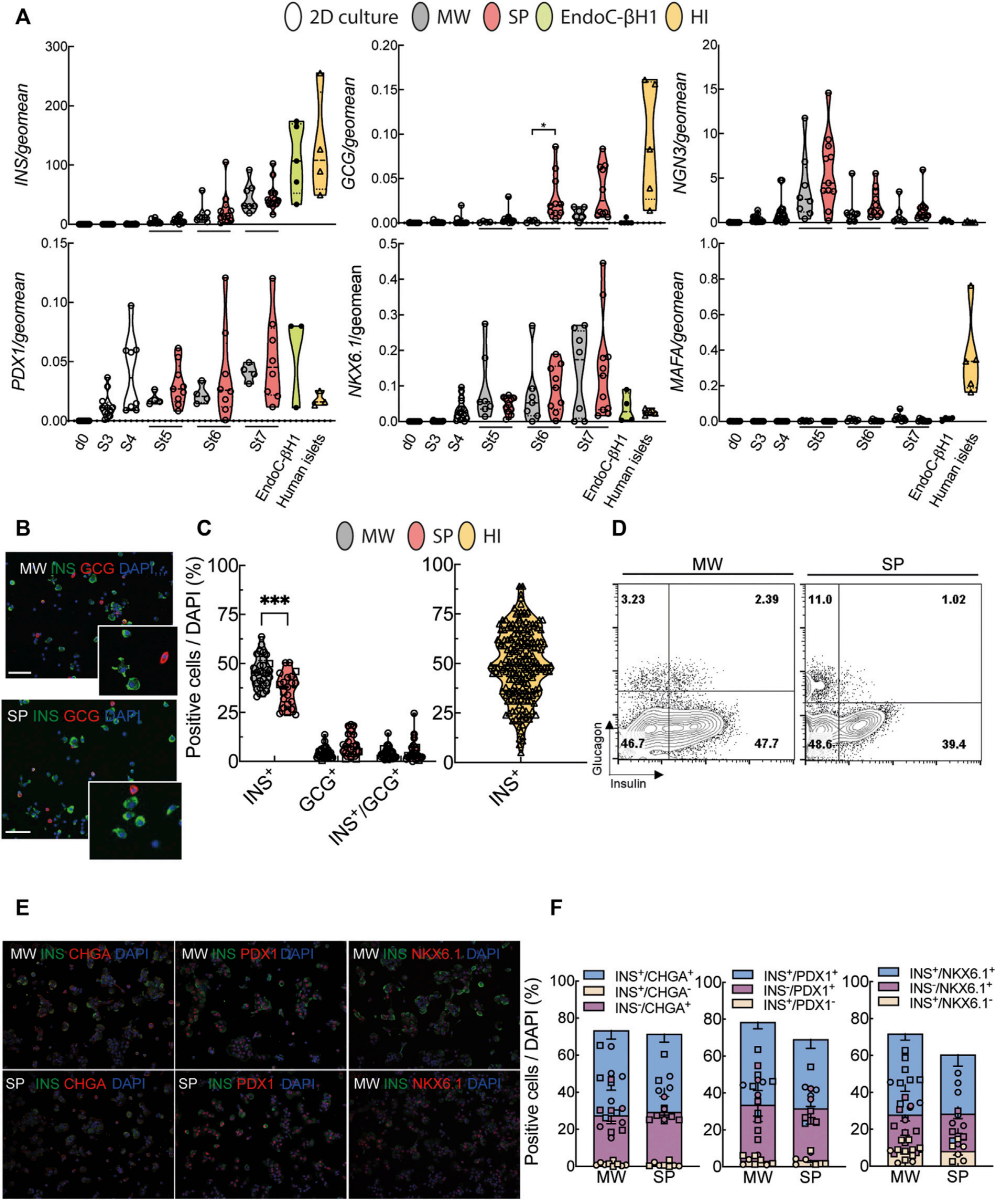
Characterization of human iPSC-derived islet-like organoids. **(A)**, Differentiation markers were measured by quantitative RT-PCR. White bars represent cells until stage 4 (St4, 2D culture), grey bars represent microwell aggregates (MW), red bars suspension aggregates (SP), green bars EndoC-βH1 cells and yellow bars human islets (HI). **(B)**, Representative pictures of dispersed aggregates stained for insulin (green) and glucagon (red). Nuclei are visualized with DAPI (blue). **(C)**, Quantification of insulin (INS), glucagon (GCG) and insulin/glucagon (INS/GCG) expressing cells from immunochemical analysis (MW n = 29; SP n = 22, HI n = 205). **(D)**, Representative flow cytometry analysis of dispersed MW and SP aggregate cells. **(E)**, Representative pictures of dispersed aggregate cells stained for insulin (green) and chromogranin A (CHGA), PDX1 or NKX6.1 (all in red). **(F)**, Quantification of immunochemical analyses of cells expressing insulin only (yellow), chromogranin A, PDX1 or NKX6.1 only (purple), and both (blue) (MW n = 8–11; SP n = 6–7). Median (bold dotted line) and quartiles (light dotted line) are shown and dots represent independent iPSC-beta differentiations or human islet preparations. Symbols represent different cell models (Hel115.6, circles; 1023A, squares, EndoC-βH1, black circles; human islets, triangles). N, number of independent experiments, defined as one iPSC-beta cell differentiation or one human islet preparation, shown as individual data points. Mixed-effects analysis followed by Bonferroni correction for multiple comparisons, *p < 0.05, **p < 0.01, ***p < 0.005.

Human iPSCs were differentiated into beta cells using a stepwise protocol (Figure 1A). Cells differentiated successfully into definitive endoderm (St1) as shown by morphology and expression of definitive endoderm marker SOX17 (Supplementary Figure S2A). Cells progressed through primitive gut tube, posterior foregut and formed pancreatic progenitors (St4) co-expressing PDX1 and NKX6.1 (Supplementary Figure S2A). At this point cells were transferred from monolayer culture into rotating suspension or static microwells to sustain the formation of islet-like aggregates. 7 million cells were plated per low attachment 6-well in 5 ml for suspension, whilst the optimal cell density for microwells was tested by seeding 500, 750, 1,000 and 2,000 cells/microwell in 2 ml and monitoring aggregate diameters. At lower cell densities (500–750 cells/microwell), aggregates kept a stable diameter of 103 ± 1 μm during culture, while at higher densities aggregate size increased with time (Figures 1B,C). This increase was not due to cell proliferation, since very few Ki67-positive cells were detected (Figure 1D), but due to fusion of aggregates that were displaced to neighboring microwells. Based on these data, we selected 750 cells/microwell for further experiments. Suspension aggregates also increased in size between St5 and St7 due to fusion. Forty-50% of the suspension differentiations were lost due to such massive clustering. Similar results were obtained with the iPSC line 1023A. Worse outcomes were observed using iPSC line ULBi.001.BJ.6 and embryonic stem cell line H1, for which suspension culture resulted in clumping and loss of the experiments (n = 5 and n = 3, respectively). In contrast, the success rate of microwell experiments was 91–100% for these stem cell lines, pointing to greater inter-cell line reproducibility (Supplementary Figure S4A). The suspension aggregates were larger (369 ± 6 μm at St6) and much more heterogeneously sized (range 231–606 μm, Figure 1B). Some of the aggregates generated in rotating suspension culture showed a dense core of DAPI-stained nuclei (Supplementary Figure S3C) suggesting central necrosis; this was not encountered with microwell culture. Using 3D microscopy in suspension aggregates, expression of the differentiation marker PDX1 was confirmed (Supplementary Figures S3A,B). At the end of St7, both microwell and suspension aggregates contained insulin- and PDX1-or insulin- and NKX6.1-double positive cells (Figure 1E, Figures 2E,F).

Cells followed a normal developmental pathway along the differentiation by transiently expressing endocrine progenitor marker *NGN3* at St5, both in suspension and microwell aggregates (Figure 2A). Expression of beta cell-specific transcription factors *PDX1* and *NKX6.1* started at St3 and increased progressively (Figure 2A). The mature beta cell marker *MafA* increased slightly upon differentiation but remained far below the expression of adult human islets (Figure 2A). At the end of differentiation, suspension and microwell aggregates were composed of a mixture of beta, alpha and bihormonal cells quantified by immunofluorescence and FACS (Figures 2B–D, Supplementary Figure S4B). Microwell and suspension aggregates contained 70–72% chromogranin A-positive cells, of which 42–46% were also insulin-positive (Figures 2E,F). Microwell aggregates contained 5–10% more insulin-positive cells and fewer glucagon-positive cells than suspension aggregates. The yield of insulin- and glucagon-double positive cells was similar, below 7% (Figures 2B–D). The yield of insulin-positive cells in microwells was on average comparable with the beta cell proportion in human islets from >200 donors, but much less variable (Figure 2C). Reproducibility within iPSC lines was assessed by calculating the coefficient of variation for beta cell yield. This showed more reproducible results for microwell than suspension aggregates; consistency was lower for human islets (Supplementary Table S7, Supplementary Figure S4B).

Taken together, microwells provide a more efficient, user-friendly platform to create reproducible and standardized size-controlled aggregates with similar differentiation efficiency compared to traditional suspension culture.

### Human iPSC-derived beta cells have an immature functional phenotype *in vitro*

**FIGURE 3:**
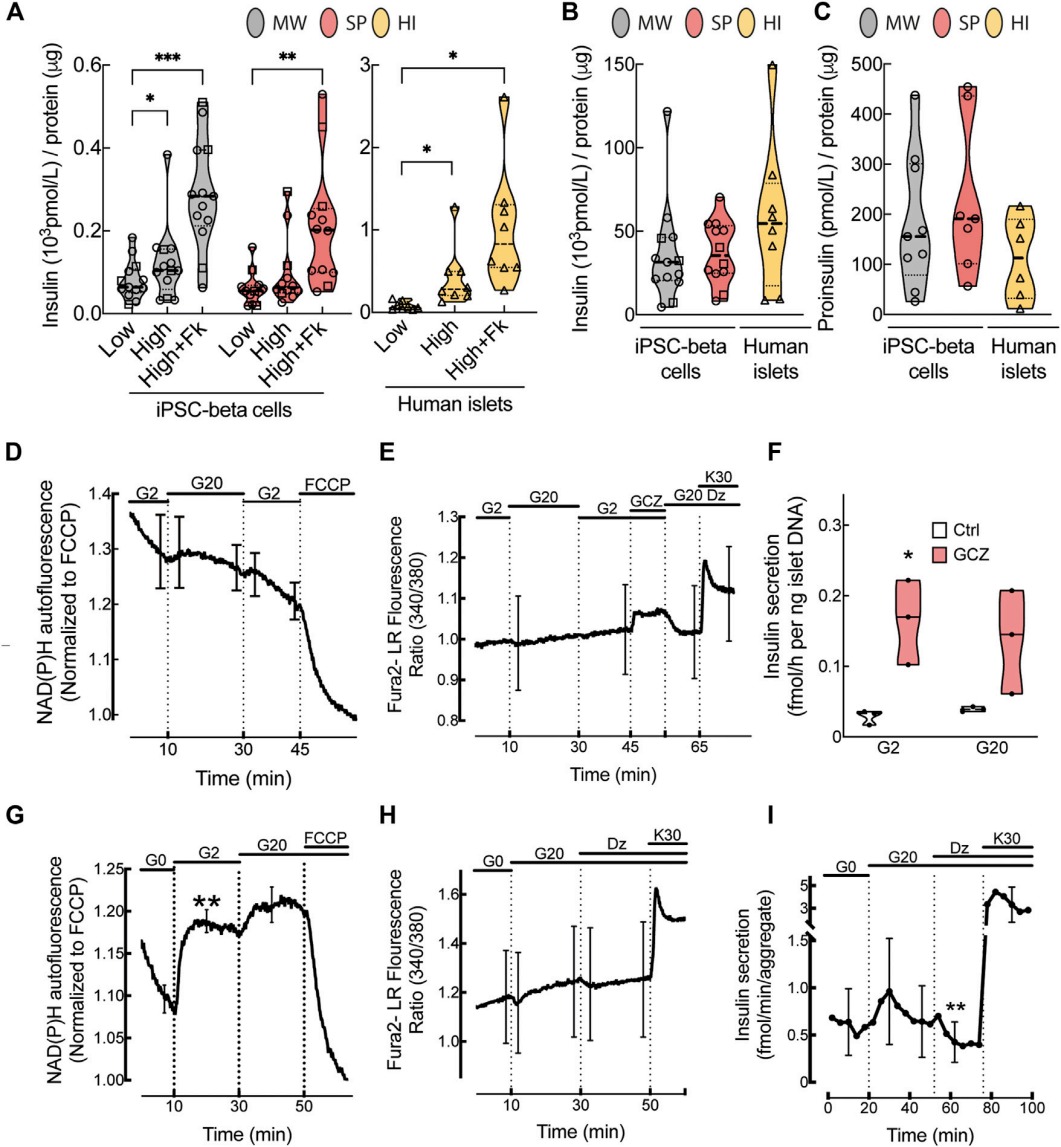
*In vitro* function of human iPSC-derived beta cells. **(A)**, Glucose-stimulated insulin release, **(B)**, insulin content (pmol/L) normalized by total protein (μg) and **(C)**, proinsulin content (pmol/L) normalized by the total protein (μg) from microwell (MW, grey bars) or suspension aggregates (SP, red bars) or human islets (HI, yellow bars). To stimulate insulin secretion, aggregates were exposed to low (1.6 mmol/L) or high (16.7 mmol/L) glucose or high glucose plus forskolin (1 μmol/L) (MW n = 13; SP n = 12; HI n = 6–8). **(D)**, NAD(P)H autofluorescence during perifusion of MW aggregates at glucose concentrations (Gn, n mmol/L), as indicated on top of the figure (n = 6). **(E)**, Fura-2 LR fluorescence ratio during perifusion of MW aggregates with KRB containing Gn and added compounds (Dz, 250 μmol/L diazoxide; GCZ, 25 μmol/L gliclazide; K30, 30 mmol/L extracellular K^+^). Data are means ± SEM for 7 preparations, each with 1-2 aggregates of each kind. **(F)**, Insulin secretion in response to G2 or G20 with or without 25 μmol/L GCZ (n = 3). **(G,H)**, Glucose-induced changes in NAD(P)H autofluorescence and fura-2 LR fluorescence ratio after 2-h glucose starvation (n = 5–6). **(I)**, Insulin secretion by glucose-starved MW aggregates during perifusion with Krebs containing G0, G20, Dz or K30 as indicated (n = 4). **(A–C)**, Median (bold dotted line) and quartiles (light dotted line) are shown; dots represent independent iPSC-beta differentiations, and symbols different cell models (Hel115.6, circles; 1023A, squares; human islets, triangles). N, number of independent experiments, defined as one iPSC-beta cell differentiation or one human islet preparation, as indicated. Mixed-effects analysis followed by Bonferroni correction for multiple comparisons, *p < 0.05, **p < 0.01, ***p < 0.005 or exact *p* value shown.

We compared *in vitro* function of microwell and suspension aggregates at St7 of differentiation. High glucose (16.7 mmol/L) stimulation elicited a modest increase (1.4 ± 0.4-fold) in insulin secretion in microwell aggregates, whilst no increase was observed in suspension aggregates (Figure 3A). Forskolin, which boosts insulin release by increasing intracellular cAMP levels, markedly stimulated insulin secretion in both (4.3 ± 1.9- and 3.1 ± 1.1-fold, respectively). Basal insulin secretion was similar, but the response to high glucose and/or forskolin was substantially below that of human islets (5.4 ± 2.5- and 13.0 ± 4.9-fold, Figure 3A). This was not due to lesser insulin content, which was in the same range for microwell aggregates and human islets and tended to be lower in suspension aggregates (Figure 3B). Reproducibility within iPSC lines was assessed by calculating the coefficient of variation for insulin secretion measures. This showed overall more reproducible results for microwell than suspension aggregates; consistency tended to be lower for human islets (Supplementary Table S8, Supplementary Figure S4C). Proinsulin content was similar in iPSC-beta cell aggregates and human islets (Figure 3C).

We further assessed functional maturation of microwell aggregates by measuring the acute effect of glucose and K_ATP_ channels modulators on NAD(P)H autofluorescence, [Ca^2+^]_i_ and insulin secretion. In non-starved aggregates, NAD(P)H autofluorescence progressively decreased during perifusion at 2 mmol/L glucose and presented a small non-significant increase upon glucose stimulation while it rapidly decreased upon mitochondrial uncoupling with FCCP (Figure 3D). In these aggregates, the sulfonylurea gliclazide, but not glucose, increased [Ca^2+^]_i_ or insulin secretion (or tended to do so) (Figures 3E,F), indicating that these aggregates have functional K_ATP_ channels.

As human beta cell lines have better glucose-stimulated insulin secretion after glucose starvation (Ravassard et al., 2011), we glucose-starved microwell aggregates for 2 h (Figures 3G–I). Under these conditions, stimulation with glucose markedly increased NAD(P)H autofluorescence, with ∼80% of maximum effect already observed at 2 mmol/L glucose. High glucose also tended to increase [Ca^2+^]_i_ and insulin secretion, and this was reversed by the K_ATP_ channel opener diazoxide.

The St5-7 culture media contain a high glucose concentration (20 mmol/L), known to exert glucotoxic effects in human islets (Weir and Bonner-Weir, 2004). In order to evaluate whether this affects St7 aggregate function, the iPSC-beta cell differentiation in microwells was tested at 5.5 mmol/L glucose, at which glucose responsiveness of human islets is better preserved during culture (Eizirik et al., 1992), and at 8 mmol/L glucose, slightly above the half-maximal effective glucose concentration of human islet insulin secretion (Henquin et al., 2006). This glucose effect was tested in basal 3 and CMRL medium, a culture medium commonly used in human islets (Murdoch et al., 2004). There was no difference in expression of beta cell markers (Supplementary Figure S5A) or yield (Supplementary Figures S5B–D) between basal 3 and CMRL media and the three glucose concentrations (5.5, 8 and 20 mmol/L). There was no improvement in NAD(P)H or [Ca^2+^]_i_ responses to glucose or other secretagogues without or with glucose starvation (Supplementary Figures S6A–D and Supplementary Figures S7A–D). Accordingly, culture of St7 aggregates at different glucose concentrations did not markedly affect insulin release in response to high glucose or KCl (Supplementary Figures S6E,F and Supplementary Figures S7E,F). Insulin secretion seemed better for non-starved aggregates cultured in Basal 3 *vs.* CMRL medium due to lower secretion at low glucose (Supplementary Figures S6E,F), while it seemed better during perifusion of glucose-starved aggregates cultured in CMRL *vs.* Basal 3 medium due to higher secretion at high glucose (Supplementary Figures S7E,F). Altogether, these data show that *in vitro* beta cell differentiation protocols reliably generate islet-like structures that lack a fully functional phenotype. Maturation is not improved by different glucose concentrations.

### Human iPSC-derived beta cells acquire a mature functional phenotype *in vivo*

**FIGURE 4:**
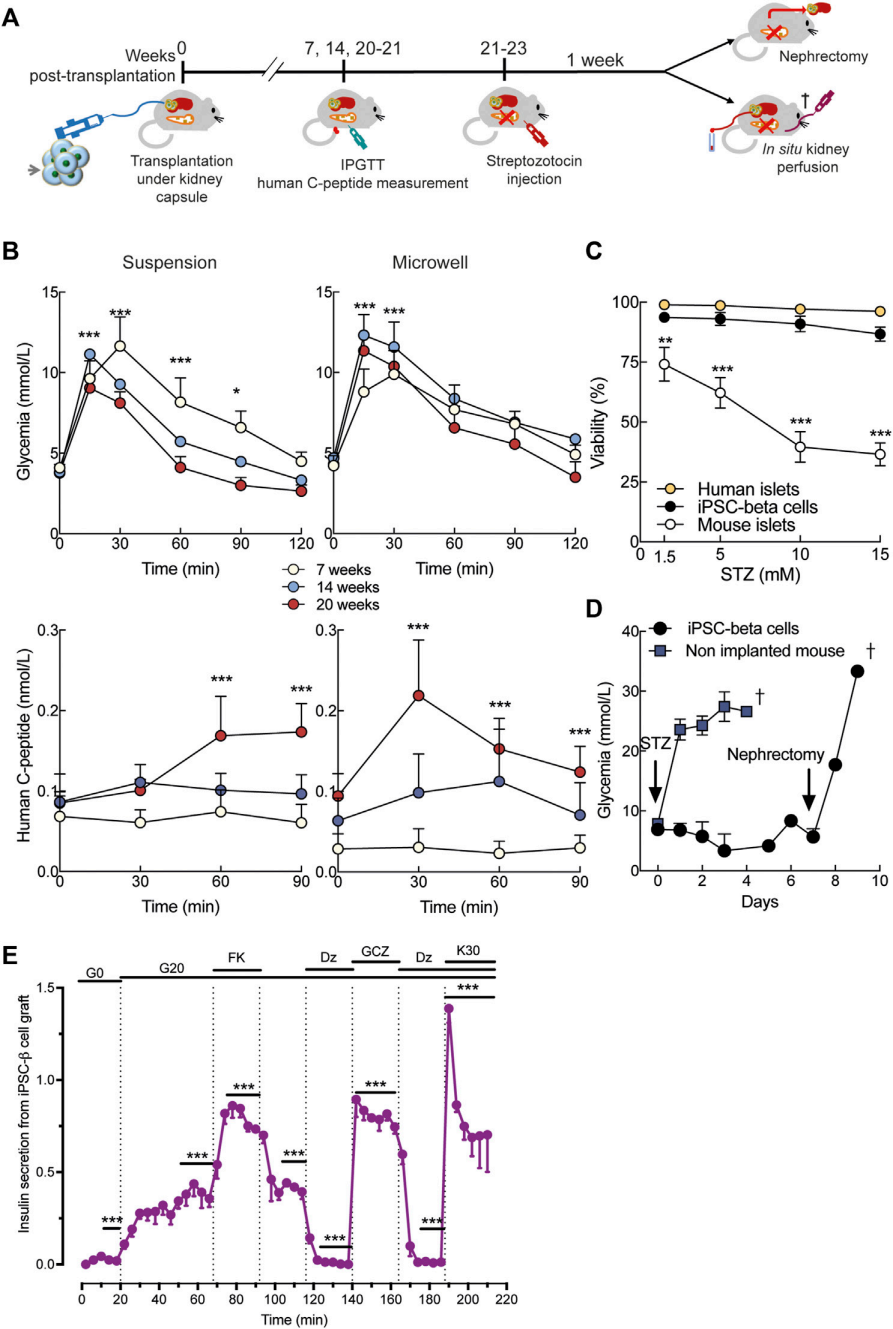
*In vivo* function of human iPSC-derived beta cells. **(A)**, Stage 7 microwell or suspension aggregates were transplanted under the kidney capsule of NOD-SCID mice. Intraperitoneal glucose tolerance test (IPGTT) was done at week 7, 14 and 20. At 21–22 weeks, mice were injected with streptozotocin (STZ) to selectively ablate mouse beta cells. Glycemia was recorded for 1 week before nephrectomy or *in situ* kidney perfusion. **(B)**, Mouse glycemia (top panels) and human plasma C-peptide (bottom panels) during IPGTT in suspension (left panels) and microwell (right panels). **(C)**, Dose-response of streptozotocin toxicity in human islets (n = 3), human iPSC-derived beta cells (n = 5) and mouse islets (n = 5) after 24-h exposure to the drug. Mixed-effects analysis followed by Bonferroni’s correction for multiple comparisons, **p < 0.01, ***p < 0.005. **(D)**, Twenty-one weeks after transplantation, a single dose of streptozotocin (200 mg/kg) was administered intraperitoneally. Non-implanted mice rapidly develop diabetes (blue, n = 5). Mice transplanted with stage 7 aggregates remain normoglycemic until graft removal by nephrectomy (black, n = 5). **(E)**, Kidney perifusion was performed 22 weeks after transplantation in two mice STZ-injected and one mouse non-STZ-injected. The grafted kidney was perifused with medium containing 0 (G0) or 20 mmol/L glucose (G20). Forskolin (1 μmol/L, FK), gliclazide (25 μmol/L, GCZ), diazoxide (250 μmol/L, DZ) and KCl (30 mmol/L, K30) were added. Secretion from each graft was normalized to the maximum secretion during the first 12-min in K30-Dz. N, number of independent experiments, defined as one iPSC-beta cell differentiation or one human or mouse islet preparation or one humanized mouse, as indicated. **p < 0.01, ***p < 0.001 *vs* basal condition (time 0-min) using mixed-effects analysis followed by Dunnett’s (b) or Sidak’s (e) correction for multiple comparison.

*In vivo* iPSC-derived cells receive differentiation and maturation cues that are lacking *in vitro*. Given the overall similar efficiency in differentiation, but difference in size of microwell and suspension aggregates, we transplanted 1,000 suspension or 3,000 microwell St7 aggregates under the kidney capsule of immunodeficient NOD/SCID mice (Figures 4A, Supplementary Figure S2B). In these humanized (i.e., transplanted with human iPSC-derived beta cells) mice, mouse glycemia and human C-peptide levels were measured in IPGTTs 7, 14 and 20 weeks post-implantation (Figure 4B). Starting from 14 weeks, microwell and suspension iPSC-beta cells acquired glucose responsiveness, as shown by glucose-stimulated human C-peptide release (Figure 4B). In parallel, mouse glucose tolerance tended to improve (Figure 4B). Streptozotocin is known to ablate selectively rodent but not human beta cells (Eizirik et al., 1994). We tested the sensitivity of human iPSC-beta cells to streptozotocin *in vitro*. Streptozotocin dose-dependently reduced viability of mouse islets, but not of human beta cells derived from iPSCs or adult organ donors (Figure 4C). At 21–23 weeks after transplantation, streptozotocin was injected intraperitoneally (Figure 4A). Non-implanted mice immediately became severely hyperglycemic, but mice with human iPSC-beta cell grafts maintained normoglycemia (Figure 4D). Nephrectomy of the graft-bearing kidney rendered the mice severely diabetic, demonstrating that the human iPSC-beta cells had provided glycemic control. Immunohistochemical analysis of grafts from streptozotocin-injected mice showed the presence of human beta and alpha cells (Supplementary Figures S2C,D).

Using *in situ* kidney perfusion, we performed detailed human beta cell functional studies *in vivo* (Figure 4A). High glucose strongly stimulated insulin secretion from the human graft and this effect was amplified by forskolin (Figure 4E). The stimulatory effect of glucose was abolished by diazoxide, restored by gliclazide and maximally stimulated by high potassium.

In sum, the *in vivo* environment boosted functional maturation of human iPSC-beta cells with acquisition of excellent responsiveness to glucose and other secretagogues.

## Discussion

Since the initial reports of successful differentiation of human iPSCs into beta cells (Pagliuca et al., 2014; Rezania et al., 2014), a body of studies has optimized differentiation strategies (Nair et al., 2019; Velazco-Cruz et al., 2019; Li et al., 2020; Yoshihara et al., 2020). However, poor differentiation reproducibility in iPSC lines continues to hamper the field. Here we describe a 3D microwell system that represents a straightforward, highly reproducible method to differentiate iPSCs into similarly sized beta cell organoids with nearly 100% experimental success rate, thereby increasing cost-effectiveness. We used three different human iPSC lines and one embryonic stem cell line and compared them to the gold standard human islets. Overall reproducibility of key results (beta cell yield and insulin secretion) was better for microwell than suspension culture. Human islets showed greater variability, which is not unexpected considering the known heterogeneity of this organ donor tissue. We also performed detailed *in vitro* and *in vivo* iPSC-beta cell functional studies. The *in vitro* function of St7 microwell aggregates was non-inferior to suspension aggregates. Both aggregate types have the machinery to secrete insulin, and respond to increased cAMP concentrations (forskolin), K_ATP_ channel modulators and high K. However, they remain poorly glucose-responsive. Only in the *in vivo* environment following transplantation did the aggregates acquire a mature phenotype comparable to human islets.

Human iPSC-beta cells represent an important technology for the modeling of beta cell pathophysiology in diabetes. Even if a good proportion of insulin-expressing cells can be achieved (40–50% in our hands), full *in vitro* functional maturation remains unfulfilled. Most differentiation protocols use monolayer culture until pancreatic progenitor stage, and then transfer cells into 3D to sustain the formation of islet-like structures (Rezania et al., 2014; Balboa et al., 2019; Liu et al., 2021). This promotes the organization of the aggregates’ cytoarchitecture. Suspension culture does not allow to control aggregate size, leading to generation of large and heterogenous structures. The bigger size results in more limited access of the cells to nutrients, growth factors and oxygen in the core, contributing to hypoxia and central necrosis. Transferring cells in 3D using microwells instead allowed to control the number of cells per aggregate, thus generating homogeneous aggregates which are analogous in size to medium-sized human islets. A comparison of techniques to generate 3D aggregates of rat islet cells, i.e. hanging drop, suspension and Sphericalplate 5D microwells, showed controlled organoid size with microwells and hanging drop cultures, with low throughput for the latter (Wassmer et al., 2020). Microwells are suitable to scaling-up; the Sphericalplate 5D was developed as a 3D culture product for human islet transplantation. Commercially available microwell plates can contain up to 35,400 aggregates per 6-well plate (AggreWell400) or they can be produced using silicon molds or 3D printing. Most beta cell differentiation protocols use high glucose concentration (20 mmol/L) in the later stages to elicit beta cell development (Pagliuca et al., 2014; Rezania et al., 2014; Balboa et al., 2018). However, chronic exposure to high glucose is known to be toxic to human islets. Culture of St7 aggregates at lower glucose concentrations did not alter expression of beta cell markers nor their function. Similarly, the two basal media we used did not alter *in vitro* glucose responsiveness. We observed adequate K_ATP_ channel activity with stimulation of calcium influx and insulin secretion by gliclazide. *In vitro* high glucose-stimulated insulin secretion was limited, however, as were changes in NAD(P)H or [Ca^2+^]_i_. This glucose response was improved after 2-h glucose starvation, but even under these conditions, aggregate metabolism was almost maximally stimulated by as little as 2 mmol/L glucose. This high glucose sensitivity is not due to a putative glucotoxic effect of the medium containing 20 mmol/L glucose, as it was also observed after 8–14-days culture at 5.5 or 8 mmol/L glucose. Longer culture at low glucose concentrations might be required in order to achieve further *in vitro* maturation (Balboa et al., 2022).

A progressive and complete functional maturation was observed *in vivo* after human iPSC-beta cell transplantation. This was demonstrated by glucose-stimulated human C-peptide secretion and maintenance of normoglycemia by human beta cell grafts after murine beta cell ablation. Of note, grafts displayed finely regulated and more mature insulin secretion in response to glucose, forskolin, diazoxide, gliclazide or KCl in the *in situ* kidney perfusion studies compared to *in vitro* static or dynamic insulin secretion patterns. It is challenging but of great interest to understand which critical factors sustain functional maturation of human iPSC-beta cells in the *in vivo* environment. Endothelial cells have been proposed to play a key role, as they are fundamental to embryonic beta cell development and adult beta cell survival (L ammert et al., 2001; Staels et al., 2019). Using single-cell transcriptomic profiling, Augsornworawat and colleagues showed that pluripotent stem cell-derived beta cells transcriptionally acquire similarity to adult beta cells following *in vivo* engraftment, with enhanced expression of mature beta cell markers (Augsornworawat et al., 2020).

We show how to generate homogeneously size-controlled islet-like organoids from human iPSCs with remarkable experimental success rate. These iPSC-derived beta cells acquire fully mature functional characteristics typical of adult human islets following *in vivo* transplantation in immunodeficient mice. The present report and future studies will eventually accomplish full *in vitro* differentiation of iPSCs into beta cells and accelerate the generation of humanized mouse models, thereby creating valuable tools for diabetes disease modelling, drug testing and cell replacement.

## Data Availability

The original contributions presented in the study are included in the article/Supplementary Material, further inquiries can be directed to the corresponding authors.
