# Supplementary material for: In depth functional characterization of human induced pluripotent stem cell-derived beta cells in vitro and in vivo
Source: Front Cell Dev Biol. 2022 Aug 17;10:967765. doi: 10.3389/fcell.2022.967765 (PMC9428245; doi:10.3389/fcell.2022.967765)
Supplement: Supplementary file 1 [file DataSheet1.pdf]

frontiers

Supplementary Material

In depth functional characterization of human induced pluripotent stem cell-derived beta cells *in vitro* and *in vivo*

Federica Fantuzzi[1,2†*], Sanna Toivonen[1†], Andrea Alex Schiavo[1], Heeyoung Chae[3], Mohammad Tariq[3], Toshiaki Sawatani[1], Nathalie Pachera[1], Ying Cai[1], Chiara Vinci[1], Enrico Virgilio[1], Laurence Ladriere[1], Mara Suleiman[4], Piero Marchetti[4], Jean-Christophe Jonas[3], Patrick Gilon[3], Décio L. Eizirik[1], Mariana Igoillo-Esteve[1], Miriam Cnop[1,5*]

[1]ULB Center for Diabetes Research, Université Libre de Bruxelles, Brussels, Belgium;

[2]Endocrinology and Metabolism, Department of Medicine and Surgery, University of Parma, Parma, Italy;

[3]Institut de Recherche Expérimentale et Clinique, Pôle d'Endocrinologie, Diabète et Nutrition, Université Catholique de Louvain, Brussels, Belgium;

[4]Department of Clinical and Experimental Medicine, University of Pisa, Pisa, Italy;

[5]Division of Endocrinology, Erasmus Hospital, Université Libre de Bruxelles, Brussels, Belgium

[†] These authors have contributed equally to this work and share first authorship

*** Correspondence:**
Miriam Cnop
mcnop@ulb.ac.be

Federica Fantuzzi
federica.fantuzzi@ulb.be

1 Supplementary Methods

1.1 Beta cell differentiation, detailed protocol.

Prior to the differentiation of iPSCs, cells were expanded in 10-cm diameter Matrigel-coated dishes. When cells reached 80% confluency, iPSCs were washed with 0.5 mmol/L EDTA, dissociated in single cells by incubation with Accutase (Capricorn Scientific) for 2-5 min, pelleted (250 g for 3-min centrifugation) and resuspended at a density of 0.8-1 million cells/mL in E8 medium supplemented with 5 µmol/L ROCK inhibitor (Y-27632 dihydrochloride, StemCell technologies) prior to seeding in Matrigel-coated 6-well plates (3 mL/well). Twenty-four hours later, (day 0, definitive endoderm induction, 2D cell culture) when cells reached full confluency, they were rinsed once with PBS and 2 ml of day 0 medium was added per well.

During days 1 to 12, comprising stages St1 (days 1-3), St2 (days 4-6), St3 (days 7-8), St4 (days 9-12), until pancreatic progenitor stage (2D cell culture), 2 mL of medium was refreshed every day according to the differentiation stage (Figure 1a, Tables S2 and S3). At days 3 and 12 cells were fixed with 4% paraformaldehyde to verify by immunohistochemistry the expression of endodermal (SOX17) and pancreatic progenitor (PDX1 and NKX6.1) markers, respectively (Figure S2). At days 0, 7, 12 cell pellets were collected to assess by real-time PCR expression of beta cell differentiation markers. Briefly, cells were rinsed twice with PBS, detached using a cell scraper and stored at -80°C until RNA extraction (Main text, methods section).
At day 13, cells were transferred to 3D culture (see main text, Methods section). Cells were washed with 0.5 mmol/L EDTA, incubated with Accutase for 5-8 min at 37°C, collected and pelleted by 3-min centrifugation at 250 g. Cells were resuspended in St5 medium (Tables S2 and S3) supplemented with 10 µmol/L ROCK inhibitor. They were plated either in suspension (5 mL/well) on an orbital shaker (96 rpm rotational speed) or in microwells (2 mL/well, AggreWell400, StemCell technologies).
During days 13 to 15 (St5, until endocrine precursor stage, 3D cell culture), half (1 mL/microwell) or all of the medium (5 mL/suspension well) was refreshed every day. A cell pellet was also collected at day 15.
From day 15 until the end of the differentiation, corresponding to stages St6 (6-7 days) and St7 (7-8 days, maturing beta cells in 3D cell culture), half (1 mL/microwell) or all of the medium (5 mL/suspension well) was refreshed every other day and cell pellets were collected at days 22 and 31. The last days of St7 islet-like aggregates were dispersed and seeded on a chamber slide to quantify the proportion of insulin- and glucagon-positive cells by immunocytochemistry.
Basal media composition and specific stage media are described in Supplementary Tables S1 and S2.

1.2 Sample size

Sample size was calculated using G*Power software version 3.1. Based on a pilot study we predict a standard deviation of 3.05 mmol/L in blood glucose levels during the IPGTT. Hence, to detect a difference of 5 mmol/L glucose between time 0 and 30 min after the glucose injection in animals transplanted with iPSC-derived beta cells, we need 9 mice (n=9) to obtain 90% power with a significant threshold of 0.05. For human C-peptide levels secreted, we expect a standard deviation of 0.1 mmol/L, requiring 13 mice (n=13) to obtain a p value of <0.05 with a power of 90% for a difference of 0.4 ng/ml.

2 Supplementary Tables

Table S1. Human islet characteristics

**Islet preparation**	**1**	**2**	**3**	**4**	**5**	**6**	**7**	**8**	**9**
**Unique identifier**	25/6	25/7	25/11	25/14	25/18	25/23	25/45	25/49	25/67
**Donor age (years)**	75	77	60	60	68	66	69	50	80

**Donor sex (M/F)**	M	M	F	F	M	M	F	F	M
**Donor BMI (kg/m²)**	23.1	26.1	29	23.9	NA	28.4	33.1	25.4	26.2
**Origin/source of islets[b]**	Pisa, Italy	Pisa, Italy	Pisa, Italy	Pisa, Italy	Pisa, Italy	Pisa, Italy	Pisa, Italy	Pisa, Italy	Pisa, Italy
**Donor history of diabetes?**	No	No	No	No	No	No	No	No	No
**Donor cause of death**	Cerebral hemorrhage	Trauma	Cerebral hemorrhage	Cerebral hemorrhage	Cerebral hemorrhage	Cerebral hemorrhage	Postanoxic encephalopathy	Cerebral hemorrhage	Cardiovascular disease
**Estimated purity (%)**	72	67	89	68	24	65	31	45	36

Table S2. Basal media composition

**Basal 1**	MCDB131 (Life Technologies), 2 mmol/L GlutaMAX (Life Technologies), 1.5 g/L $NaHCO_3$ (Merck Millipore), 0.5% BSA fraction V (Sigma-Aldrich), 10 mmol/L glucose (Sigma-Aldrich)
**Basal 2**	MCDB131 (Life Technologies), 2 mmol/L GlutaMAX (Life Technologies), 2.5 g/L $NaHCO_3$ (Merck Millipore), 2% BSA fraction V (Sigma-Aldrich), 10 mmol/L glucose (Sigma-Aldrich), 1:200 ITS-X (Thermo Fisher Scientific)
**Basal 3**	MCDB131 (Life Technologies), 2 mmol/L GlutaMAX (Life Technologies), 1.5 g/L $NaHCO_3$, 2% BSA fraction V (Sigma-Aldrich), 20 mmol/L glucose (Sigma-Aldrich), 1:200 ITS-X (Thermo Fisher Scientific), 10 µg/mL heparin (STEMCELL Technologies), 10 µmol/L zinc sulfate (Sigma-Aldrich), 1X penicillin streptomycin (Lonza)

Table S3. Stage-specific media composition

**D0**	Basal 1, 100 ng/mL Activin A (PreproTech), 5 µmol/L CHIR-99021 (Axon Medchem)
**D1**	Basal 1, 100 ng/mL Activin A (PreproTech), 0.5 µmol/L CHIR-99021 (Axon Medchem)
**D2**	Basal 1, 100 ng/mL Activin A (PreproTech)
**St2**	Basal 1, 0.25 mmol/L L-ascorbic acid (Sigma-Aldrich), 50 ng/mL FGF7 (PreproTech)
**St3**	Basal 2, 0.25 mmol/L ascorbic acid (Sigma-Aldrich), 50 ng/mL FGF7 (PreproTech), 0.25 µmol/L SANT-1 (Sigma-Aldrich), 1 µmol/L retinoic acid (Sigma-Aldrich), 100 nmol/L LDN-193189 (Selleckchem), 200 nmol/L TPB (Santa Cruz)

**St4**	Basal 2, 0.25 mmol/L L-ascorbic acid (Sigma-Aldrich), 50 ng/mL FGF7 (PreproTech), 0.25 µmol/L SANT-1 (Sigma-Aldrich), 0.1 µmol/L retinoic acid (Sigma-Aldrich), 200 nmol/L LDN-193189 (Selleckchem), 100 ng/mL EGF (STEMCELL Technologies), 10 mmol/L nicotinamide (Sigma-Aldrich), 10 ng/mL Activin A (PreproTech)
**St5**	Basal 3, 0.05 µmol/L retinoic acid (Sigma-Aldrich), 0.25 µmol/L SANT-1 (Sigma-Aldrich), 100 nmol/L LDN-193189 (Selleckchem), 1 µmol/L GC-1 (Tocris), 100 nmol/L GSiXX (Merck Millipore), 10 µmol/L ALK5 inhibitor II (ENZO), 20 ng/mL betacellulin (PreproTech)
**St6**	Basal 3, 100 nmol/L LDN-193189 (Selleckchem), 10 µmol/L ALK5 inhibitor II (ENZO), 1 µmol/L GC-1 (Tocris), 100 nmol/L GSiXX (Merck Millipore)
**St7**	Basal 3, 10 µmol/L ALK5 inhibitor II (ENZO), 1 µmol/L GC-1 (Tocris), 10 µmol/L Trolox (Sigma-Aldrich), 20 µmol/L SP600125 (Selleckchem), 75 µmol/L resvetratrol (Sigma-Aldrich), 2 µmol/L R428 (STEMCELL Technologies), 1 mmol/L N-acetyl-cysteine (Sigma-Aldrich)

Table S4. Sequence of primers used for real-time PCR

**Human gene**	**Forward sequence (5’→3’)**	**Reverse sequence (5’→3’)**
GAPDH	CAGCCTCAAGATCATCAGCA	TGTGGTCATGAGTCCTTCCA
ACTB	CTGTACGCCAACACAGTGCT	GCTCAGGAGGAGCAATGATC
MAFA	GCCAGGTGGAGCAGCTGAA	CTTCTCGTATTTCTCCTTGTAC
PDX1	AAAGCTCACGCGTGGAAA	GCCGTGAGATGTACTTGTTGA
NKX6.1	GGGCTCGTTTGGCCTATT	CGTGCTTCTTCCTCCACTT
NEUROD1	CTATCACTGCTCAGGACCTACT	CCACTCTCGCTGTACGATTT
INS	CCAGCCGCAGCCTTTGTGA	CCAGCTCCACCTGCCCCA
GCG	GCTAAACAGAGCTGGAGAGTA T	AAGCCCTCTTTGGGAACTT
NGN3	GACGACGCGAAGCTCACCAA	TACAAGCTGTGGTCCGCTAT

Table S5. Antibodies used for immunocytochemistry.

Rabbit anti-human OCT4	Cell Signaling Technology Cat# 2840, RRID:AB_2167691	1:400
Goat anti-human SOX17	R and D Systems Cat# AF1924, RRID:AB_355060	1:500
Mouse anti-human NKX6.1	BD Biosciences Cat# 563022, RRID:AB_2737958	1:250
Goat anti-human PDX1	R and D Systems Cat# AF2419, RRID:AB_355257	1:500

Guinea pig anti-human insulin	Agilent Cat# A056401-2, RRID:AB_2617169	N/A
Mouse anti-human glucagon	Sigma-Aldrich Cat# G2654, RRID:AB_259852	1:1000
Rabbit anti-human nucleoli antibody	Abcam Cat#ab190710, RRID:NA	1:300
Mouse anti-human chromogranin A	Agilent Cat#M0569, RRID: AB_2081135	1:500
Rabbit anti-human vimentin	Abcam Cat#137321, RRID:NA	1:800
Mouse anti-human betaIII tubulin	Promega Cat#G7121, RRID:AB_430874	1:500
Mouse anti-human SSEA-4	Thermofisher Scientific, Cat#MA1-021, RRID: AB_2536687	1:500
Mouse anti-human TRA-1-60	Thermofisher Scientific, Cat#MA1-023, RRID: AB_2536699	1:100
Rabbit anti-human Nanog	Cell Signalling, Cat#4903, RRID:10559205	1:400
Alexa Fluor® 488 AffiniPure Donkey Anti-Mouse IgG (H+L)	Jackson ImmunoResearch Laboratories Cat#715-545-151, RRID:AB_2341099	1:500
Rhodamine Red™-X (RRX) AffiniPure Donkey Anti-Goat IgG (H+L)	Jackson ImmunoResearch Laboratories Cat#705-295-147, RRID:AB_2340423	1:500
Alexa Fluor® 488 AffiniPure Donkey Anti-Guinea Pig IgG (H+L)	Jackson ImmunoResearch Laboratories Cat#706-545-148, RRID:AB_2340472	1:500
Rhodamine Red™-X (RRX) AffiniPure Donkey Anti-Mouse IgG (H+L)	Jackson ImmunoResearch Laboratories Cat#715-295-151, RRID:AB_2340832	1:500
Alexa Fluor® 647 AffiniPure Donkey Anti-Goat IgG (H+L)	Jackson ImmunoResearch Laboratories Cat#705-605-147, RRID:AB_2340437	1:500
Rhodamine Red™-X (RRX) AffiniPure Donkey Anti-Rabbit IgG (H+L)	Jackson ImmunoResearch Laboratories Cat#711-295-152, RRID:AB_2340613	1:500
Alexa Fluor® 488 AffiniPure Donkey Anti-Rabbit IgG (H+L)	Jackson ImmunoResearch Laboratories Cat#711-545-152, RRID:AB_2313584	1:500

Table S6. Antibodies used for flow cytometry.

Rabbit anti-insulin Alexa Fluor 647 conjugated	Cell Signaling Technology; Cat# 3014S	1:50
Mouse anti-glucagon BV421 conjugated	BD, Cat#565891	1:50

Table S7. Coefficients of variation for the beta cell yield in different pluripotent stem cell lines at St7.

		Microwells		Suspension		Human islets	
		CV (%)	n	CV (%)	n	CV (%)	n
INS^+	Hel115.6	18	22	24	15		
	1023A	17	5	24	5		
	ULBi.001.BJ.6	22	6	*	*		
	H1	35	3	*	*		
	Human islets					34	201
INS^+/ $NKX6.1^+$	Hel115.6	20	3	24	3		
	1023A	26	8	77	3		
INS^+/ $PDX1^+$	Hel115.6	12	3	6	3		
	1023A	27	5	52	3		
INS^+/ $CHGA^+$	Hel115.6	15	3	9	3		
	1023A	48	3	49	3		

Beta cell markers were insulin alone, or insulin plus NKX6.1, insulin plus PDX1 and insulin plus chromogranin A. The coefficient of variation (CV) for the yield for each cell line was calculated as ratio of the standard deviation and the mean of the indicated number (n) of independent experiments. * All differentiations failed due to clumping in suspension culture. Human islets are shown as the gold standard reference. Individual experiments for these cell lines are shown in Figure S4B.

Table S8. Coefficients of variation for insulin secretion in different pluripotent stem cell lines at St7.

		Microwells		Suspension		Human islets	
		CV (%)	n	CV (%)	n	CV (%)	n
BASAL	Hel115.6	58	10	67	9		
	1023A	64	3	71	3		
	ULBi.001.BJ.6	52	4	*	*		
	H1	60	3	*	*		
	Human islets					63	8
HIGH	Hel115.6	77	10	80	9		
	1023A	60	3	83	3		
	ULBi.001.BJ.6	52	4	*	*		
	H1	55	3	*	*		
	Human islets					88	8
SI	Hel115.6	30	10	21	9		
	1023A	11	3	14	3		
	ULBi.001.BJ.6	56	4	*	*		

	H1	13	3	*	*		
	Human islets					45	8

Insulin secretion was assessed at 1.6 mM (BASAL) or 16.7 mM glucose (HIGH). The stimulation index (SI) was calculated as ratio of insulin released at HIGH and BASAL. The coefficient of variation (CV) for insulin secretion for each cell line was calculated as ratio of the standard deviation and the mean of the indicated number (n) of independent experiments. * All differentiations failed due to clumping in suspension culture. Human islets are shown as the gold standard reference. Individual experiments for these cell lines are shown in Figure S4C.

3. Supplementary Figures

Figure S1

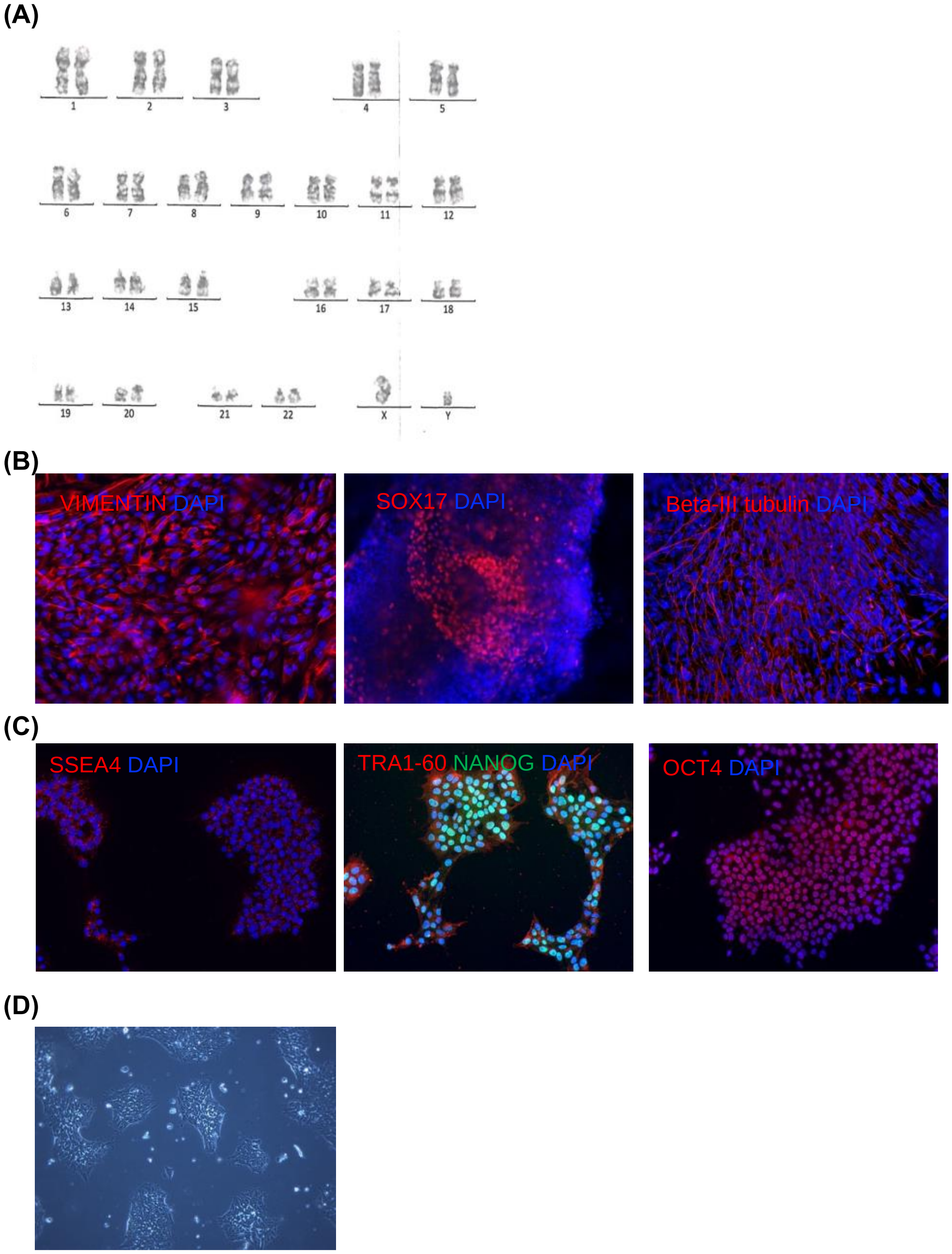

Figure S1. Characterization of iPSC line ULBi001.BJ.6 derived from healthy donor fibroblasts. A, Normal 46XY karyotype visualized with G-banding. B, Immunofluorescence analysis of embryoid bodies. Vimentin (left, red) is used as a marker of mesoderm, beta-III-tubulin (center, red) as a marker of ectoderm and SOX17 (right, red) as an endoderm marker. Nuclei are visualized with DAPI. C, Immunofluorescence for pluripotency markers SSEA4 (left, red), TRA1-60 (center, red) and NANOG (center, green) and OCT4 (right, red). Nuclei are visualized with DAPI. D, Morphology by bright field microscopy of ULBi001.BJ.6 iPSC colonies.

Figure S2

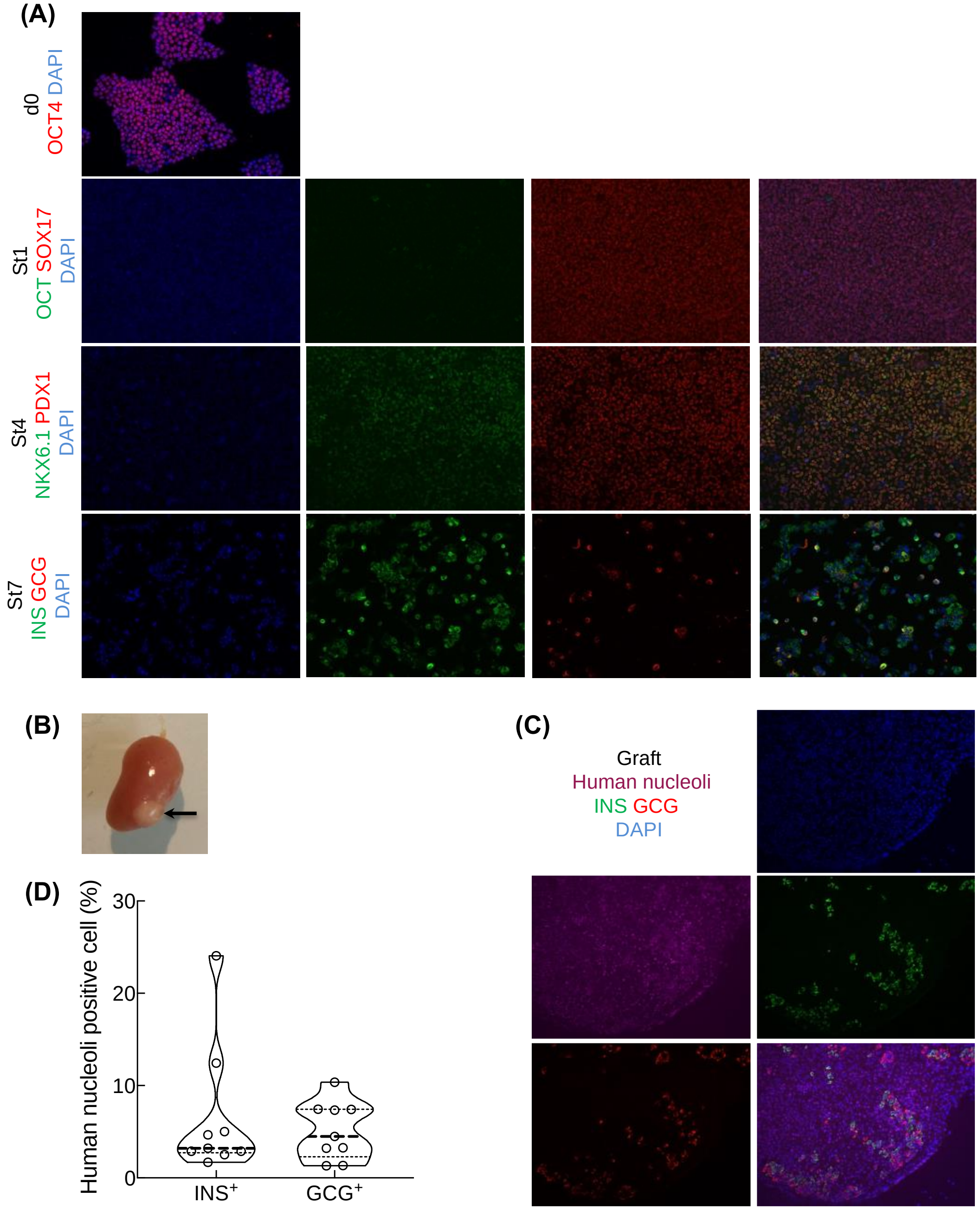

Figure S2. Characterization of Hel115.6 cells during *in vitro* differentiation and after transplantation. A, Representative pictures of iPSCs across the differentiation stained for the pluripotency marker OCT4 (red) at day 0 (d0) and stage (St) 1 (green), for the endodermal marker SOX17 (red) at St1 and for the pancreatic progenitor markers NKX6.1 (green) and PDX1 (red) at St4. At the end of the differentiation (St7) the majority of cells are stained for insulin (INS, green) and fewer cells are positive for glucagon (GCG, red). Nuclei are visualized with DAPI (blue). B, Representative picture of the graft under the mouse kidney capsule 22 weeks post-transplantation. C, Representative pictures and D, quantification of iPSC-derived cells in the retrieved graft stained for insulin (INS, green), glucagon (GCG, red) and human nucleoli (pink). Nuclei are visualized with DAPI (blue) (n=9).

Figure S3

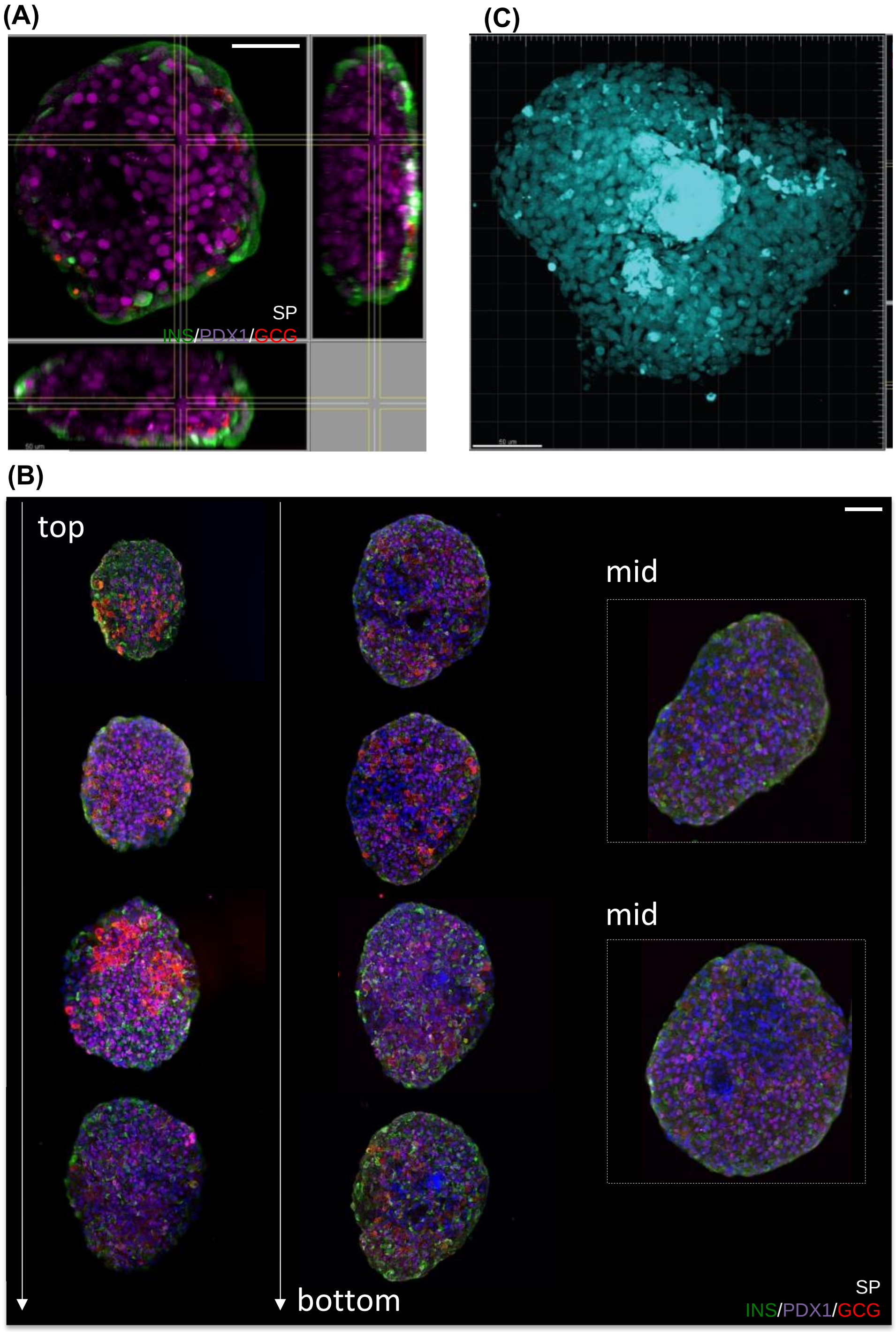

Figure S3. Characterization of aggregates cultured in suspension. A-B, Representative pictures of iPSC-derived cells cultured in suspension and stained for insulin (INS, green), glucagon (GCG, red) and PDX1 (pink). Nuclei are visualized with DAPI (blue). B, Sequential pictures acquired with confocal microscopy show the cytoarchitecture of one representative aggregate from the top to the bottom layer of the spherical structure. Additional mid-aggregate sections are shown in the right panels. C, Representative picture of a larger aggregate cultured in suspension. Nuclei are stained with DAPI (sky blue). The dense core suggests presence of central necrosis.

Figure S4

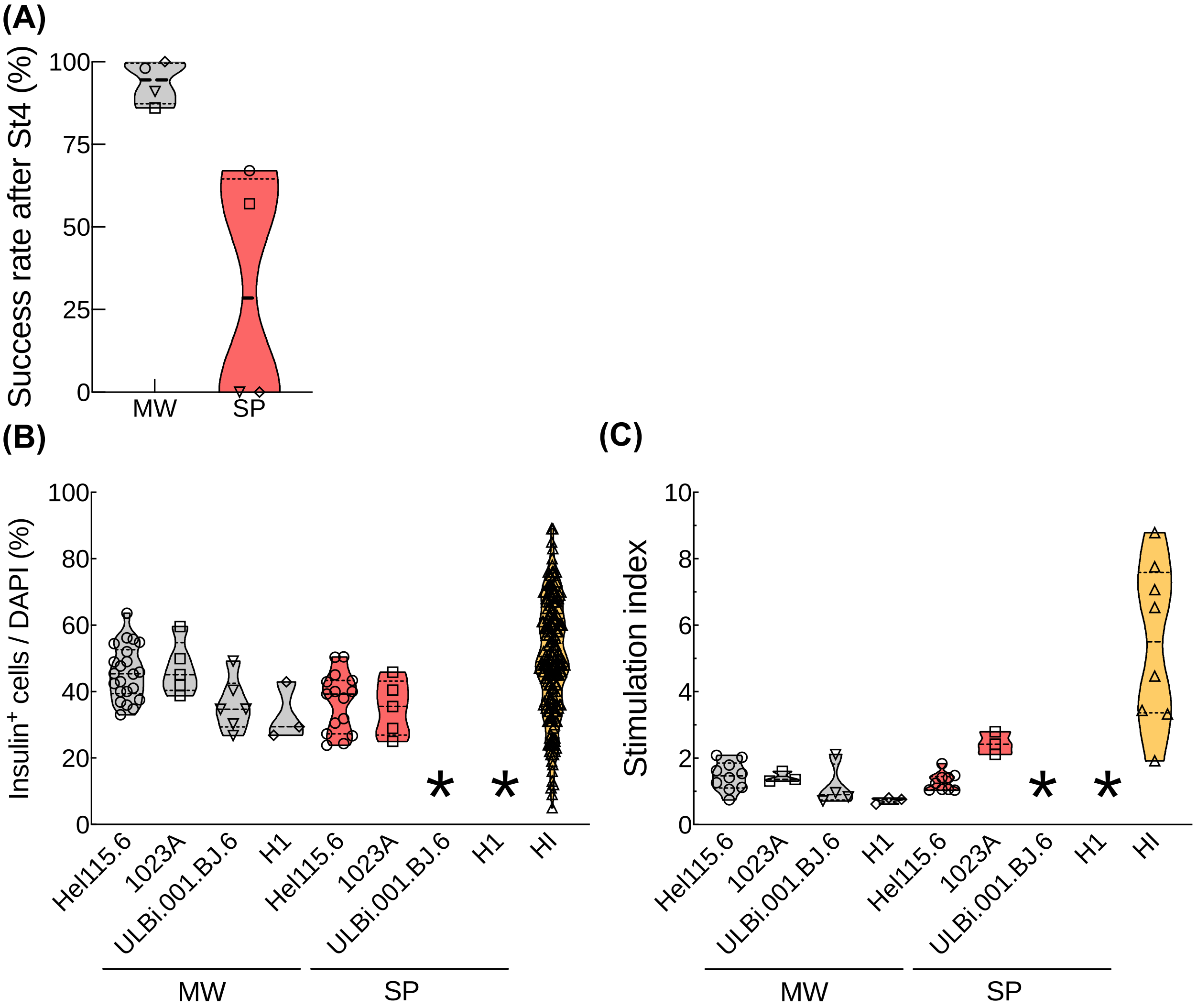

Figure S4. Intra- and inter-cell line reproducibility of the differentiation. A, Reproducibility between cell lines for the success rate of differentiations done in parallel in microwell (MW) versus suspension culture (SP), i.e. differentiations that successfully reached the end. Circles represent Hel115.6 cell line (n=3), squares 1023A (n=7), inverted triangles ULBi.001.BJ.6 (n=5) and diamonds H1 (n=3). Reproducibility in terms of B, yield of insulin-positive cells (quantified by immunocytochemistry) and C, glucose-responsive insulin secretion (stimulation index calculated as ratio of insulin release at 16.7 versus 1.6 mM glucose) in different pluripotent stem cell lines. Circles represent Hel115.6 (B, MW n=22 and SP n=15; C, MW n=10 and SP n=9), squares 1023A (B, MW and SP n=5; C, MW and SP n=3), inverted triangles ULBi.001.BJ.6 (B, MW n=6 and SP n=0; C, MW n=3 and SP n=0) and diamonds H1 (B, MW n=3 and SP n=0; C, MW n=3 and SP n=0). * All differentiations failed due to clumping in suspension culture. Human islets are shown as the gold standard reference (HI, triangles, B, n=201, C, n= 8).

Figure S5

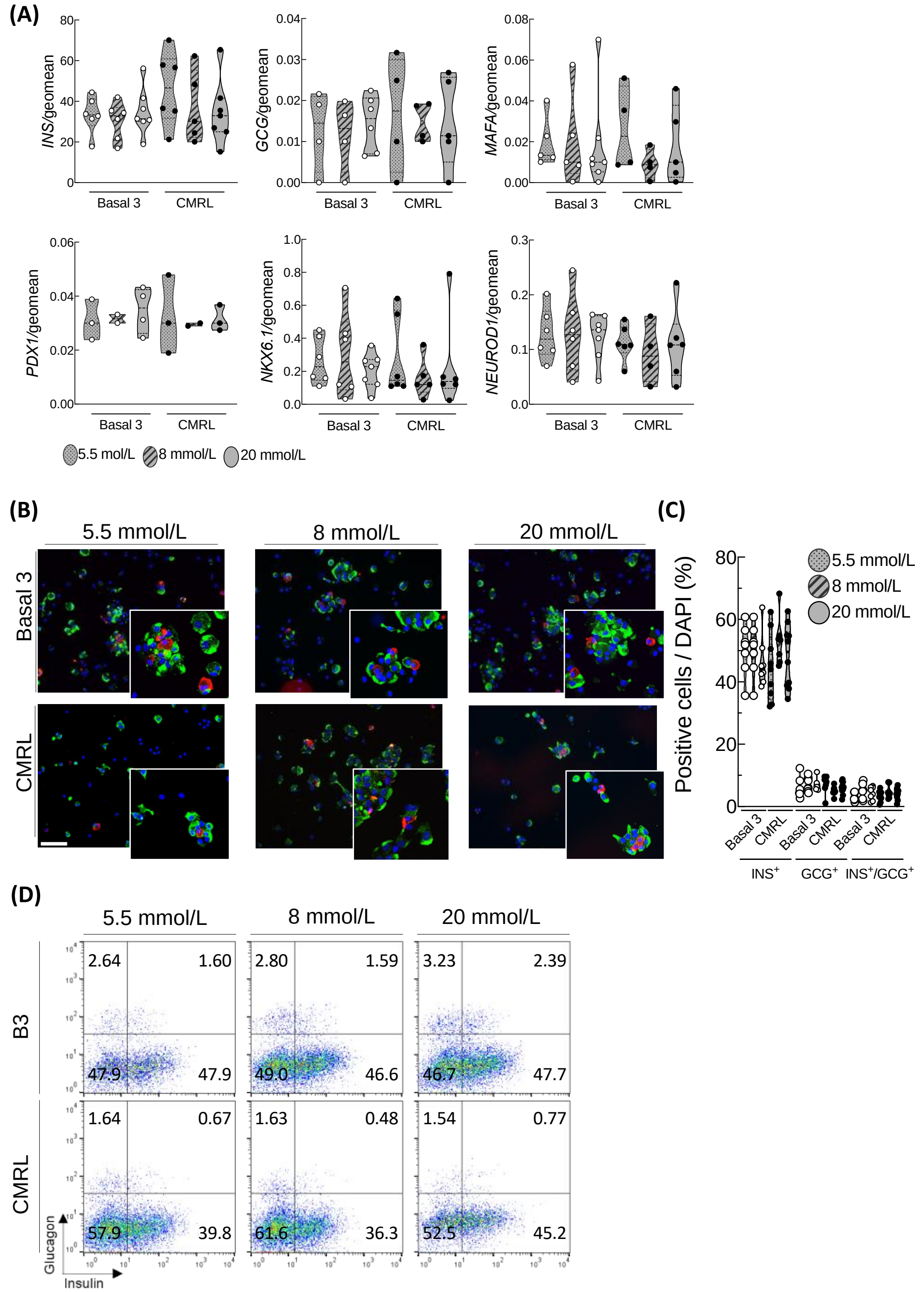

Figure S5. Quantitative RT-PCR of microwell iPSC-beta cells cultured in glucose concentrations 5.5 (dotted gray bars), 8 (striped gray bars) or 20 mmol/L (plain gray bars) and basal 3 (white circles) or CMRL media (black circles). A, Differentiation markers were measured by quantitative RT-PCR. (n=2-7). B, Representative pictures and C, quantification of dispersed aggregates stained for insulin (INS, green) and glucagon (GCG, red). Nuclei are visualized with DAPI (blue) (n=7-10). D, Representative flow cytometry analysis of dispersed microwell aggregates. Median (bold dotted line) and quartiles (light dotted line) are shown and dots represent independent experiments.

Figure S6

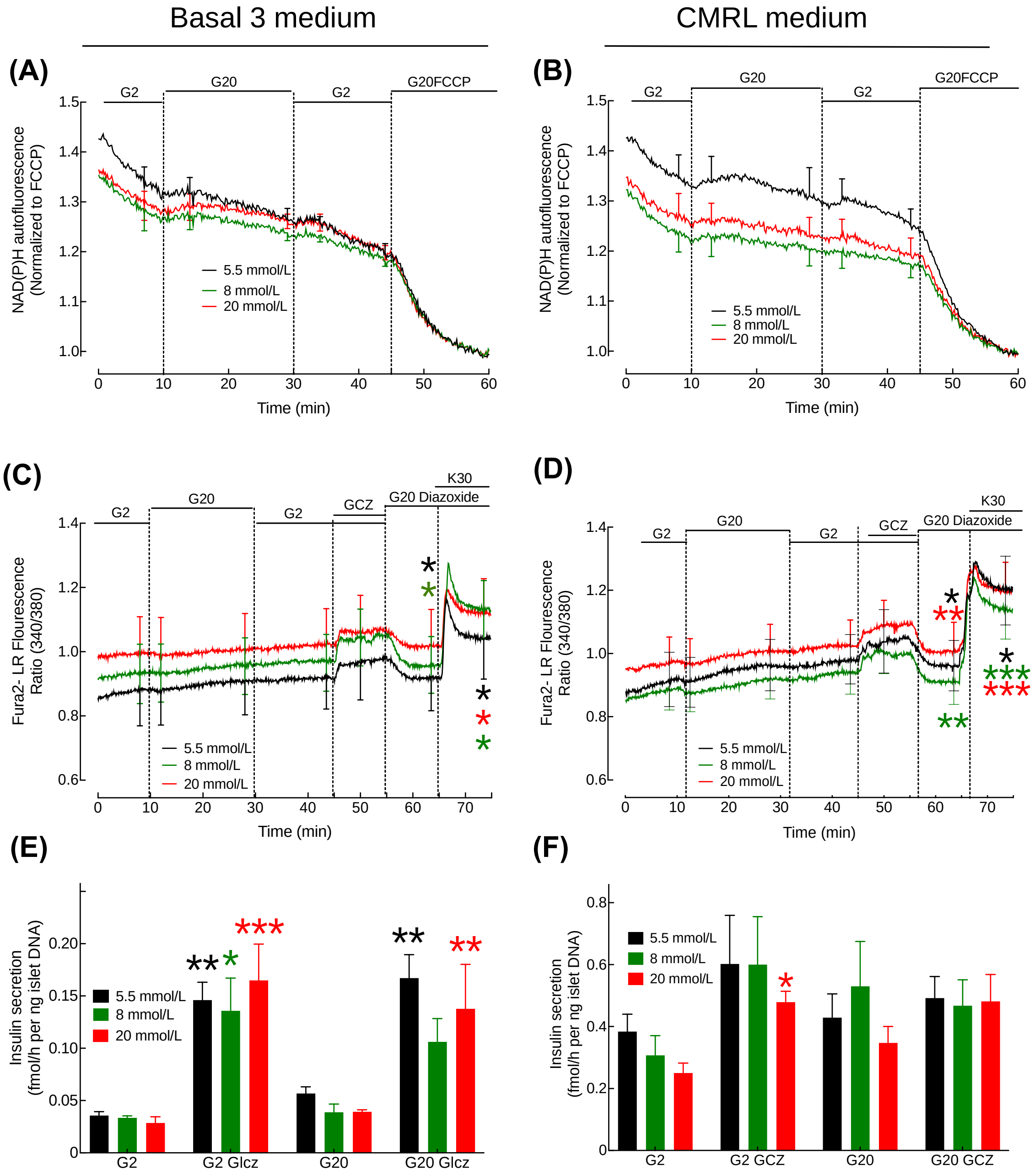

Figure S6. Effect of culture medium and glucose concentration on *in vitro* function of human iPSC-derived beta cells. St7 microwell aggregates were cultured 10-14 days in basal 3 (A, C, E) or CMRL medium (B, D, F) containing 5.5 (black trace), 8 (green trace) or 20 (red traces) mmol/L glucose before measurement of their acute responses to glucose and other secretagogues. Changes in NAD(P)H autofluorescence, $[Ca^{2+}]_i$ and insulin secretion were assessed as in Fig. 3(d-i). (A-B) Data are means ± SEM for 4 (G5) or 6 (G8 & G20) preparations, each with 1 or 2 aggregates. (C-D) Data are means ± SEM for 5 (G5), 7 (G8) or 6-7 (G20) preparations, each with 1 or 2 aggregates. (E-F) Data are means ± SEM for 3 preparations in duplicate. *$p<0.05$; **$p<0.01$, ***$p<0.001$ by one-way ANOVA (for repeated measurements when the comparison was made between selected time points in the same trace) followed by Sidak's correction for multiple comparisons

Figure S7

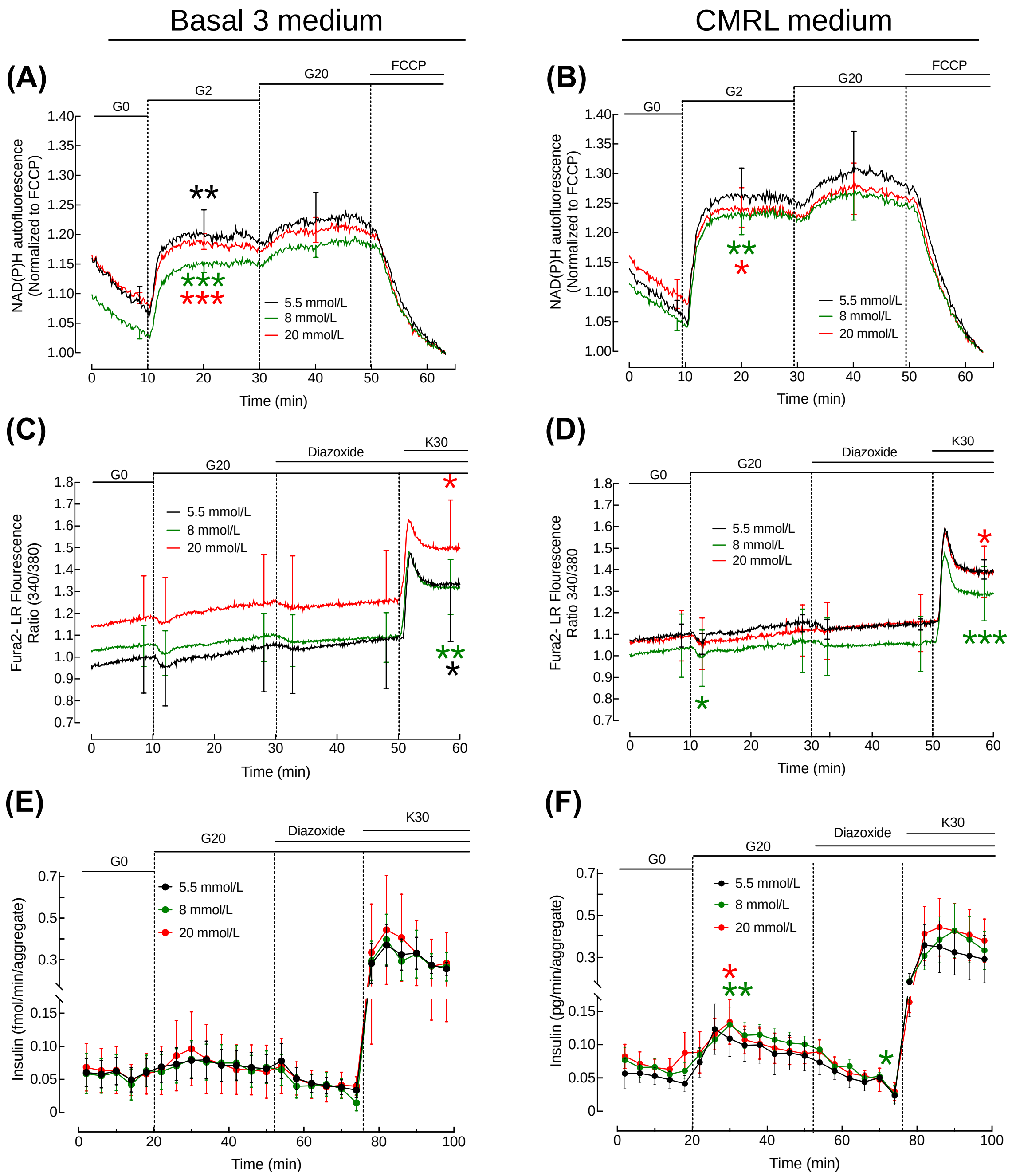

Figure S7. Effect of culture medium and glucose concentration on *in vitro* function of human iPSC-derived beta cells after 2-hour glucose starvation. St7 microwell aggregates were cultured 10-14 days in basal 3 (A, C, E) or CMRL medium (B, D, F) containing 5.5 (black trace), 8 (green trace) or 20 (red traces) mmol/L glucose before 2-hour glucose starvation followed by measurement of their acute response to glucose and other secretagogues. Changes in NAD(P)H autofluorescence, $[Ca^{2+}]_i$ and insulin secretion were assessed as in Fig. 3(d-i). (A-B) Data are means ± SEM for 4 (G5), 6 (G8) or 5 (G20) preparations, each with 1 or 2 aggregates. (C-D) Data are means ± SEM for 3 (G5), 5 (G8), or 4 (G20) preparations, each with 1 or 2 aggregates. (E-F) Data are means ± SEM for 4 preparations. Statistical analysis as in ESM Fig. 5.
